# Multicenter evaluation of anatomical landmark versus technology-assisted techniques for chemotherapy venous access device implantation

**DOI:** 10.1038/s41598-026-49241-4

**Published:** 2026-09-01

**Authors:** Nicolas Felipe Camargo, Arnold Barrios, Jorge Vargas, Danilo Chaparro, Juan Barriga, Alvaro Ariza, Cielo Uscategui, Diana Munetones

**Affiliations:** 1 General Surgery, Fundación Universitaria Sanitas, Bogotá, Colombia; 2 General Surgery, Clínica Reina Sofía, Bogotá, Colombia; 3 Gastrointestinal Surgery, Clínica Universitaria Colombia, Bogotá, Colombia; 4 Epidemiology Department, Universidad del Rosario, Bogotá, Colombia; 5 Radiology, Clínica Los Cobos, Bogotá, Colombia; 6 Medicine and Surgery, Fundación Universitaria Sanitas, Bogotá, Colombia

**Keywords:** Chemotherapy access port, Technology assessment, Port-a-cath, Totally implantable venous access device (TIVAD), Anatomic landmarks, Cancer, Diseases, Health care, Medical research, Oncology

## Abstract

The global incidence of cancer has significantly increased, resulting in the expanded use of innovative systemic treatments that require implantable vascular access devices, thus increasing the frequency of device implantation. These implants are associated with low complication rates, good patient tolerance, and a relatively short learning curve for both surgeons and radiologists. However, various factors, including patient characteristics, anatomical variations, underlying pathologies, and the surgeon’s preferred technique, continue to pose challenges to standardized implementation. This variability may influence procedural success and complication rates. Therefore, a systematic comparison between anatomical landmark-based and technology-assisted approaches is necessary to determine whether differences in outcomes are attributable to the implantation method itself and to support evidence-based clinical decision-making. Hence, the importance of evaluating the outcomes of two common approaches to device implantation: one based on anatomical landmarks, relying solely on surface anatomical references for vascular access, and the other assisted by technology, which incorporates real-time imaging modalities such as ultrasound guidance for venous cannulation and fluoroscopic imaging for catheter tip positioning and procedural verification. This retrospective, multicenter cohort study analyzed oncology patients indicated for intravenous chemotherapy, with data collected from four institutions in Bogotá between June 1, 2016, and December 31, 2021. A total of 867 patients with diagnoses of hematolymphoid, gastric, colon, pancreatic, or breast cancer were included, excluding those with hypercoagulability syndrome, arrhythmias, or pacemaker use at the time of implantation. The data were systematically recorded using the REDCap^®^ application. Quantitative variables were analyzed using descriptive statistics, whereas categorical variables were analyzed using the chi-square test or Fisher’s exact test, as appropriate. Continuous variables were assessed using *t* tests or Mann–Whitney U tests. Kaplan‒Meier survival models were used to analyze the time to complications based on the catheter insertion technique, and the anatomical reference point (ARP) technique was compared with the technology-assisted (TA) insertion technique. The incidence of complications was calculated using the relative risk (RR) with a significance level of *p* < 0.05. A total of 3,308 clinical records of patients undergoing totally implantable venous access device (TIVAD) implantation were reviewed, and 867 individuals who met the inclusion criteria were selected: 464 (53.5%) in the technology-assisted (TA) group and 403 (46.5%) in the anatomical reference point (ARP) group, with equivalent sociodemographic characteristics. The statistically significant findings included the presence of hematomas as a complication and the use of prophylactic antibiotics and fluoroscopy to assess catheter positioning, both of which are associated with a higher complication rate at 90 days post-operation. Additionally, a surgical time greater than or equal to 30 min was associated with a trend toward greater incidence of complications, although this difference did not reach statistical significance (RR = 4.24). No significant differences were found between implantation techniques based on anatomical reference points (ARPs) and those based on technology-assisted (TA) methods in terms of complication rates. The implantation of TIVADs in oncology patients with different primary cancers is safe and feasible. Although this study provides a clear definition of the advantages of different techniques, future research using controlled clinical trials is recommended to validate our findings and definitively determine the optimal TIVAD insertion technique.

## Introduction

The global incidence of cancer approached 18.1 million individuals in 2020, with an expected projection of 29.5 million by 2040^1^. According to the World Health Organization (WHO), a total of 113,000 new cases were recorded in Colombia in 2020^1^. Consequently, the use of innovative systemic treatment regimens, which require implantable vascular access devices, has increased, making this procedure increasingly common, considering the low complication rates, good patient tolerance, and short learning curve for surgeons^2^. However, other therapies, such as prolonged antibiotic treatments and chronic parenteral nutrition, have also benefited from these devices^3^.

Since Niederhuber in 1982, a medical device standard has been developed on the basis of nonexposed, radiopaque central vascular access, which can be punctured multiple times in a self-sealing silicone central area^4^. The implantation of devices has optimized the use of new technologies in surgical practice^5^, however, challenges remain due to the individual characteristics of each patient, anatomical variations, underlying pathologies, and even the surgeon’s traditional approach^6^.

Currently, two principal approaches are used for the implantation of totally implantable venous access devices (TIVADs). The first is the anatomical landmark-based approach (ARP), in which venous cannulation is performed using surface anatomical reference points—most commonly the internal jugular or subclavian vein—without real-time imaging guidance. The second is the technology-assisted approach (TA), which incorporates imaging modalities such as ultrasound for guided venous access and fluoroscopy and/or intracavitary electrocardiography for catheter tip positioning and procedural verification.

The choice between these approaches may be influenced by patient-specific anatomical characteristics, comorbidities, operator experience, institutional protocols, and availability of technological resources. Such variability contributes to heterogeneity in clinical practice and may affect procedural efficiency, safety, and complication rates [7]. Despite the increasing adoption of technology-assisted techniques, robust comparative evidence remains limited in Latin America, where differences in resource availability and surgical training may further impact outcomes.

To our knowledge, this study represents the largest cohort conducted in Latin America comparing anatomical landmark-based and technology-assisted techniques for TIVAD implantation. By evaluating 90-day postoperative complications, this study aims to provide region-specific evidence to clarify whether the implantation method itself influences clinical outcomes and to support evidence-based decision-making in oncologic vascular access management.

## Materials and methods

A retrospective, multicenter cohort study was conducted in oncology patients indicated for intravenous chemotherapy. The procedures were performed between June 1, 2016 and December 31, 2021, at four hospitals in Bogotá, Colombia. Three of these institutions—Clínica Reina Sofía, Clínica Universitaria Colombia, and Central de Urgencias Puente Aranda—belong to the Colsanitas Clinic Group, a private healthcare network. The fourth institution, Los Cobos Medical Center, is an independent tertiary referral hospital.

The inclusion criteria comprised patients of both sexes aged 18 years or older who underwent catheter implantation at one of the four participating institutions. Eligible patients were required to have documented clinical and radiological follow-up and a diagnosis of an oncologic condition, including hematolymphoid, gastric, colon, pancreatic, or breast cancer.

Patients with known hypercoagulable disorders, documented cardiac arrhythmias, or implanted pacemakers at the time of port placement were excluded to minimize potential confounding effects related to their underlying clinical conditions. These exclusion criteria were established to reduce selection bias and ensure that postoperative outcomes were more directly attributable to the implantation technique itself rather than to complications arising from pre-existing comorbidities that could independently influence thrombotic events, catheter-related complications, or procedural risk. Patients who underwent venodisection were excluded from the comparative analysis.

Following the approval of the study protocol by the ethics committees of Colsanitas Clinic group and Los Cobos Medical Center, data collection was initiated via electronic medical records in RedCap^®^. These data were collected by attending physicians from each institution, who were previously authorized as investigators. Clinical data were extracted from SOPHIA^®^ version 6.2.7 (Keralty Group, Bogota) for inpatient records and AVICENA^®^ version 7.7.1 (Keralty Group, Bogota) for outpatient records. Imaging data were obtained via Carestream Vue Motion^®^ software (Carestream Health, Inc., based on Rochester, New York, USA), Hospital Information System (HIS): SAP^®^, and Radiology Information System (RIS): Centricity^®^ (General Electric, based in Boston, Massachusetts, USA) for Los Cobos Medical Center.

To ensure data quality, an audit was performed by cross-referencing information with the medical records of patients treated by the general surgery department and the surgical program database at each institution. To minimize missing data and biologically implausible variables, multiple data sources, including clinical records, surgical databases, and radiological systems, were used. Every ten medical records were reviewed by the principal investigator at each center to verify the accuracy and completeness of the data. The position of the catheter tip was independently assessed by three radiologists through a review of the corresponding imaging studies. A nonprobabilistic consecutive sampling method was used to select clinical records from 867 patients based on the inclusion criteria. The data were systematically recorded via the REDCap^®^ application.

### Surgical technique

The anatomical reference point–based (ARP) technique involved venous cannulation guided exclusively by surface anatomical landmarks, most commonly targeting the internal jugular or subclavian vein. After successful venous puncture, catheter placement was completed using the Seldinger technique, including guidewire insertion, vessel dilation, and advancement of the catheter into the intravascular space. Subsequently, an infraclavicular subcutaneous pocket was created for reservoir placement.

The technology-assisted (TA) technique involved percutaneous venous cannulation under real-time imaging guidance, primarily ultrasound or fluoroscopy, to access the internal jugular or subclavian vein. Imaging guidance allowed direct visualization of vascular structures and needle trajectory. Catheter advancement was subsequently performed using the Seldinger technique. The port reservoir was positioned following the same procedural approach as in the ARP technique.

### Statistical analysis

Quantitative variables were analyzed via frequency measures, central tendency, and dispersion. Normality assumptions were evaluated via the Shapiro‒Wilk nonparametric test. Categorical data were analyzed using absolute and relative frequencies. Bivariate analysis of categorical variables was conducted via the chi-square test for independence and Fisher’s exact test. For continuous variables, t tests and the Mann‒Whitney U test were applied. Kaplan–Meier survival models were used to analyze time to complication occurrence according to the technique employed. Survival curves were compared using the log-rank test. The incidence of complications associated with the catheter insertion technique was calculated via the rate ratio or relative risk (RR) and compared between practices based on anatomical reference points (ARP) in TIVAD catheter implantation and those implanted via technology-assisted (TA) methods. Hypothesis testing was considered statistically significant at p values < 0.05. The statistical analysis was performed via the R programming language version 4.2.0.

### Ethics approval and consent to participate

As this type of research was based on documentary review of medical records and performed without any intentional intervention or modification of biological, physiological, psychological, or social variable is classified as risk-free, therefore the requirement for informed consent was waived by the Research Ethics Committee of Fundación Universitaria Sanitas (Approval No. CEIFUS 1395-22). Furthermore, the present study was conducted in accordance with Colombian Resolution 8430 of 1993, Colombian Resolution 2378 of 2008, Good Clinical Practice (GCP) guidelines, the Declaration of Helsinki (Fortaleza, Brazil, 2013), and Colombian Law 1581 of 2012 regarding the protection of personal data. This study did not involve any experiments on humans or the use of human tissue samples. The research consisted exclusively of a retrospective review of existing medical records, without any direct intervention, experimental procedures, or manipulation of human participants.

## Results

### Patient characteristics

A total of 3,308 patient medical records for totally implantable venous access device (TIVAD) implantation were reviewed, and 867 individuals met the inclusion criteria, consisting of 464 (53.5%) technology-assisted (TA) technique patients and 403 (46.5%) anatomical reference points (ARP) patients, with equivalent sociodemographic populations. On average, the patients in the cohort were 55.7 years old (SD ± 14.1) and had a Body Mass Index (BMI) of 24.65 (SD ± 4.5), and the surgical time for TIVAD insertion was 49.37 min (SD ± 21.8). A greater proportion of women, 573 (66.1%), were included, as was a younger population between 18 and 64 years of age, 609 (70.2%), and a normal BMI, 445 (51.3%). Histologically, breast cancer was the most common cancer (352, 40.6%), followed by hematological cancer (188, 22.0%) in chemotherapy patients. Catheter insertion was performed on an outpatient basis in 713 (82.2%) patients across the participating institutions (Table 1). No device-associated mortality was observed, and all patients completed the 90-day post-operative follow-up.

**Table 1:** Characteristics of the population.

Characteristic	TA (*n*, %)	ARP (*n*, %)	*p* value	Total (*n*, %)
Sex				
Female	309 (35.6%)	264 (30.4%)	0.736	573 (66.1%)
Male	155 (17.9%)	139 (16.0%)		294 (33.9%)
Age (Years)				
18 to 64	323 (37.3%)	286 (33.0%)	0.616	609 (70.2%)
65+	140 (16.1%)	118 (13.6%)		258 (29.8%)
BMI (kg/m²)*				
Underweight [< 18,5]	23 (2.7%)	33 (3.8%)	0.136	56 (6.5%)
Normal [18,5–24,9]	234 (27.0%)	211 (24.3%)		445 (51.3%)
Overweight [25–29,9]	144 (16.6%)	113 (13.0%)		257 (29.6%)
Obesity [ > = 30]	64 (7.4%)	45 (5.2%)		109 (12.6%)
Preoperative Diagnosis				
Breast Cancer	194 (22.2%)	158 (18.2%)	0.575	352 (40.6%)
Hematologic Cancer	91 (10.5%)	97 (11.2%)		188 (21.7%)
Colon Cancer	80 (9.2%)	71 (8.2%)		151 (17.4%)
Gastric Cancer	78 (9.0%)	60 (6.9%)		138 (15.9%)
Pancreatic Cancer	21 (2.4%)	17 (2.0%)		38 (4.4%)
Admission Service				
Outpatient	380 (43.8%)	333 (38.4%)	0.778	713 (82.2%)
Hospitalization	84 (9.7%)	70 (8.1%)		154 (17.8%)

TA: TIVAD catheter insertion through technology-assisted practice, ARP: TIVAD catheter insertion through practice on the basis of anatomical reference points, BMI: body mass index according to WHO classification.

### Comparative analysis of methods

Antibiotic prophylaxis use was recorded for 369 (42.6%) and was significantly more likely to be used in technology assisted placement (33.1%) compared to landmark assisted placement (9.5%, *p* < 0.001). Similar results were observed with the use of local anesthesia technique for 440 (50.7%) subclavian vein access for 665 (76.7%), and right-sided lateralization for 584 (67.4%) patients, when comparing the two groups. Vascular cannulation via ultrasound guidance (466 (53.8%)), reservoir fixation (829 (95.6%)), and resident participation or assistance (233 (26.9%)) were also commonly noted (Table 2).

**Table 2:** Variable procedure for implantation of TIVADs.

Characteristics	TA *n* (%)	ARP *n* (%)	*p* value	Total *n* (%)
Prophylactic Antibiotic				
No	177 (20.4%)	321 (37.0%)	< 0.001	498 (57.4%)
Yes	287 (33.1%)	82 (9.5%)		369 (42.6%)
Anesthesia Type				
Local	277 (31.9%)	163 (18.8%)	< 0.001	440 (50.7%)
Local + Sedation	156 (18.0%)	156 (18.0%)		312 (36.0%)
General	31 (3.6%)	84 (9.7%)		115 (13.3%)
Vascular Site for Catheter Placement				
Subclavian Vein	288 (33.2%)	377 (43.5%)	< 0.001	665 (76.7%)
Internal Jugular Vein	162 (18.7%)	19 (2.2%)		181 (20.9%)
External Jugular Vein	14 (1.6%)	3 (0.3%)		17 (2.0%)
Cephalic Vein	0 (0.0%)	3 (0.3%)		3 (0.3%)
Innominate Vein	0 (0.0%)	1 (0.1%)		1 (0.1%)
Catheter Lateralization				
Right	297 (34.3%)	287 (33.1%)	0.023	584 (67.4%)
Left	167 (19.3%)	116 (13.4%)		283 (32.6%)
Vascular Cannulation Method				
Under Ultrasound Guidance	466 (53.8%)	0 (0.0%)		466 (53.8%)
Through Anatomical Landmarks	0 (0.0%)	392 (45.2%)		392 (45.2%)
Resident Assistance				
No	608 (70.1%)	25 (2.9%)		633 (73.0%)
Yes	230 (26.5%)	4 (0.5%)		234 (27.0%)
Reservoir Fixation				
No	39 (4.5%)	0 (0.00)		39 (4.5%)
Yes	799 (92.2%)	29 (3.3%)		828 (95.5%)

TA: TIVAD catheter insertion via technology-assisted practice, ARP: TIVAD catheter insertion via practice based on anatomical reference points, The location of the distal tip of the catheter was determined primarily via radiography on the basis of Cadman’s criteria (815; 94.0%). Alternatively, fluoroscopy (242; 27.9%) and EKG (26; 3.0%) were used less frequently (Table 3).

**Table 3:** Final location of the catheter tip. The determination of the distal catheter tip position via the Cadman classification was performed only initially in 815 (94.0%) patients.

Characteristic	TA *n* (%)	ARP *n* (%)	*p* value	Total *n* (%)
EKG				
No	438 (50.5%)	403 (46.5%)	< 0.001	841 (97.0%)
Yes	26 (3.0%)	0 (0.0%)		26 (3.0%)
Fluoroscope				
No	230 (26.5%)	395 (45.6%)	< 0.001	625 (72.1%)
Yes	242 (27.9%)	0		242 (27.9%)
Distal end of the catheter (Cadman)*				
Atriocaval junction	168 (20.6%)	76 (9.3%)	–	244 (29.9%)
Distal third of the superior vena cava	79 (9.7%)	54 (6.6%)		133 (16.3%)
Right atrium	66 (8.1%)	66 (8.1%)		132 (16.2%)
Middle third of the superior vena cava	51 (6.3%)	39 (4.8%)		90 (11.1%)
Inferior vena cava	26 (3.2%)	62 (7.6%)		88 (10.8%)
Proximal third of the superior vena cava	25 (3.1%)	40 (4.9%)		65 (8.0%)
Superior vena cava	17 (2.1%)	10 (1.2%)		27 (3.3%)
Brachiocephalic vein junction with superior vena cava	18 (2.2%)	18 (2.2%)		36 (4.4%)

TA: TIVAD catheter insertion assisted by technology, ARP: TIVAD catheter insertion on the basis of anatomical reference points.

### Adverse events

The primary outcome, which was complications within 90 days of catheter insertion, occurred in 29 (3.3%) patients. Among these complications, 19 (2.2%) occurred in the TA group, and 10 (1.2%) occurred in the ARP group, with no significant differences between the groups.

According to the Dindo‒Clavien severity classification, most complications were Grade I (18, 62.1%), with 22 (75.9%) occurring within the first 30 days, primarily due to pneumothorax (9, 31.0%), of which 7 occurred in the ARP group, whereas 2 occurred in the TA group. The immediate complications included 11 hematomas, nine pneumothoraxes, and 6 infections associated with the device. Except for hematoma, which is a complication of the procedure, there were no statistically significant differences between the two implantation methods in terms of the occurrence of complications (Table 4).

**Table 4:** Distribution of complications during follow-up.

Characteristic	TA *n* (%)	ARP *n* (%)	*p* value	Total *n* (%)	
Total postcatheter complications (90 days)					
No	445 (51.3%)	393 (45.3%)	0.255	838 (96.7%)	
Yes	19 (2.2%)	10 (1.2%)		29 (3.3%)	
Postcatheter complications (30 days)					
No	452 (52.1%)	394 (45.4%)	0.66	845 (97.5%)	
Yes	13 (1.5%)	9 (1.0%)		22 (2.5%)	
Complication Chronology					
Early (Less than 30 days)	13 (1.5%)	9 (1.0%)	0.366	22 (2.5%)	
Late (More than 30 days)	6 (0.7%)	1 (0.1%)		7 (0.8%)	
Dindo-Clavien					
I	13 (1.5%)	5 (0.6%)	0.1	18 (2.1%)	
II	3 (0.3%)	0 (0.0%)		3 (0.3%)	
III	3 (0.3%)	5 (0.6%)		8 (0.9%)	
Complication					
Hematoma	11 (1.3%)	0 (0.0%)	< 0.001	11 (1.3%)	
Pneumothorax	2 (0.2%)	7 (0.8%)	0.08	9 (1.0%)	
Difficulty in medication administration	6 (0.7%)	3 (0.3%)	0.51	9 (1.0%)	
Infection	4 (0.5%)	2 (0.2%)	0.69	6 (0.7%)	
Pain secondary to medication administration	4 (0.5%)	1 (0.1%)	0.37	5 (0.6%)	
Dermatological injury secondary to medication administration	5 (0.6%)	0 (0.0%)	0.06	5 (0.6%)	
Deep vein thrombosis	1 (0.1%)	0 (0.0%)	1	1 (0.1%)	
Reservoir rotation	1 (0.1%)	0 (0.0%)	1	1 (0.1%)	
Pain at insertion site	0 (0.0%)	1 (0.1%)	0.46	1 (0.1%)	

**Fig. 1:**
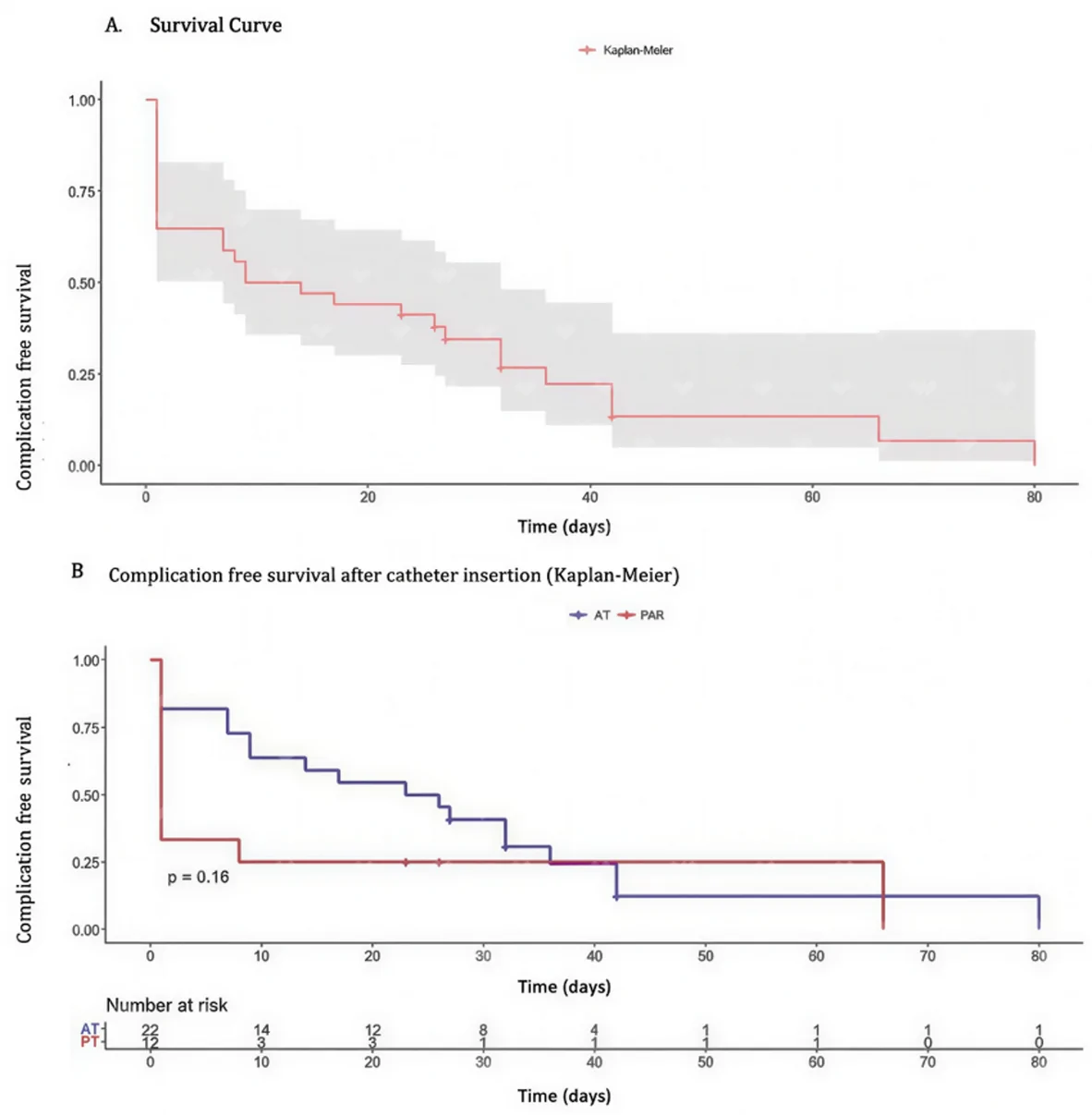
Kaplan–Meier curves of complication-free survival according to implantation technique. (**A**) overall complication-free survival, (**B**) complications by type of TIVAD catheter.

TA: insertion of the TIVAD catheter through technology-assisted practice, ARP: insertion of the TIVAD catheter through practice on the basis of anatomical reference points, The median time to the occurrence of complications at 90 days was 11.5 days (95.0% CI 7–32). For the AT group, the median duration was 24.5 days, whereas for the ARP group, it was 1 day, which was mainly due to pneumothorax. There were no statistically significant differences in complication rates between the two implantation techniques, as demonstrated by the Kaplan–Meier curves of complication-free survival, which showed comparable 90-day event-free probabilities across both groups. (log-rank test = 0.16) (Fig. 1).

An abnormal BMI, whether underweight or overweight/obese (Relative Risk (RR) 1.29; 95.0 CI 0.63–2.65), antibiotic prophylaxis use (RR 2.56; 95.0% CI 1.20–5.44), right-sided lateralization (RR 1.68; 95.0% CI 0.82–3.44), surgical time of 30 min or more (RR 4.24; 95.0% CI 0.58–30.85), and fluoroscopy use for tip positioning (RR 3.17; 95.0% CI 1.55–6.49) were associated with a greater complication rate at 90 days. However, only antibiotic use and fluoroscopy were statistically significant factors for the development of complications. A univariate analysis of factors associated with higher complication rates at 30 days was conducted, but no significant relationships were found (Table 5).

An abnormal BMI, whether underweight or overweight/obese (RR 1.29; 95% CI 0.63–2.65), right-sided lateralization (RR 1.68; 95% CI 0.82–3.44), and a surgical time of 30 min or more (RR 4.24; 95% CI 0.58–30.85) were not significantly associated with complication rates at 90 days. In contrast, antibiotic prophylaxis use (RR 2.56; 95% CI 1.20–5.44) and fluoroscopy use for tip positioning (RR 3.17; 95% CI 1.55–6.49) were significantly associated with higher complication rates. A univariate analysis of factors associated with complication rates at 30 days was also performed, with no statistically significant associations identified (Table 5).

**Table 5:** Factors determining the incidence of complications at 30 and 90 days after TIVAD insertion.

Characteristic	(RR) 30 days	95% CI	(RR) 90 days	95% CI
Male	1.11	(0.47–2.62)	0.88	(0.40–1.90)
Age 18 to 64 years	0.60	(0.26–1.39)	0.79	(0.37–1.68)
Abnormal BMI	1.26	(0.55–2.88)	1.29	(0.63–2.65)
Outpatient admission	0.46	(0.11–1.96)	0.22	(0.03–1.58)
Use of prophylactic antibiotics	1.95	(0.84–4.50)	2.56	(1.20–5.44)
General anesthesia	0.65	(0.15–2.73)	0.48	(0.12–2.01)
Right laterality (Relative to the patient)	1.72	(0.75–3.93)	1.68	(0.82–3.44)
Catheter insertion via ARP	0.80	(0.34–1.85)	0.61	(0.29–1.29)
Surgical time (30 + minutes)	–	–	4.24	(0.58–30.85)
Fluoroscopy for catheter positioning	2.15	(0.94–4.90)	3.17	(1.55–6.49)
Resident assists in procedure	0.60	(0.21–1.76)	0.43	(0.15–1.23)

TA: TIVAD catheter insertion via technology-assisted practice, ARP: TIVAD catheter insertion via anatomical landmark-based practice.

## Discussion

### Patient recruitment and general considerations

This study recruited patients with various types of primary cancer, with the largest case series consisting of breast cancer patients. These patients require special consideration, as venous access to the ipsilateral limb is avoided following surgery with axillary lymphadenectomy. This necessitates continuous adaptation of the surgeon’s skills^8^.

**Fig. 2:**
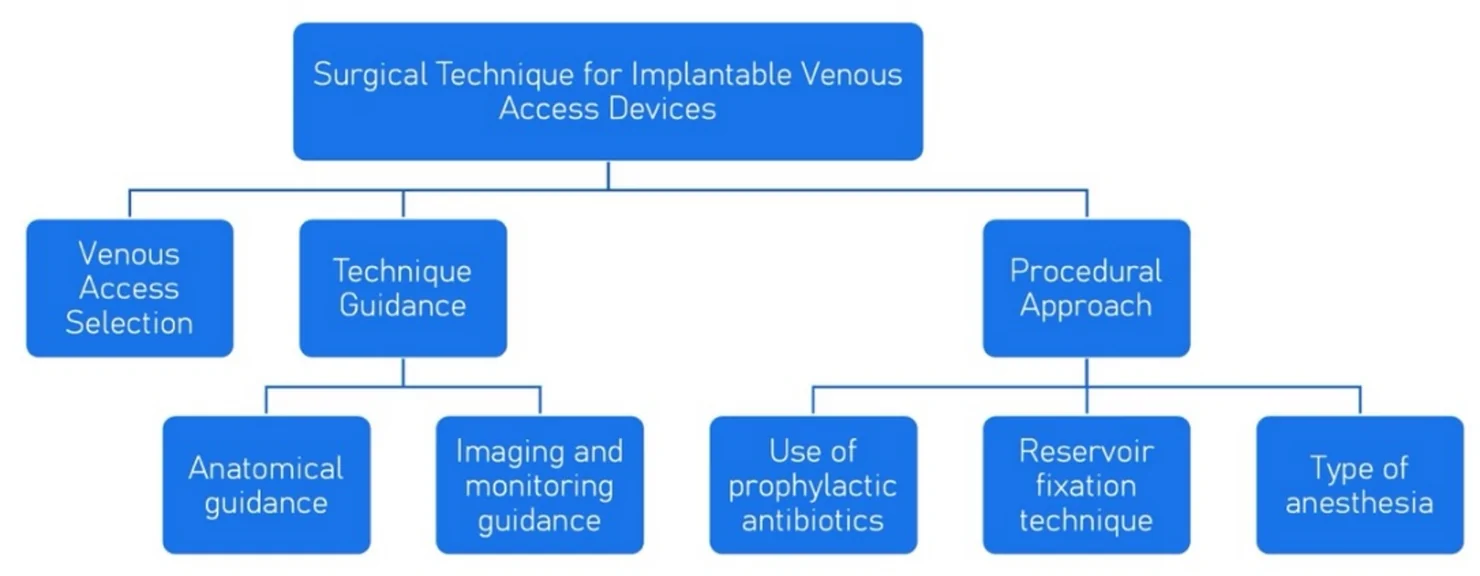
Decisions in the implementation of implantable vascular access devices.

In general, totally implantable venous access devices (TIVADs) provide a safe and comfortable route for the administration of cytotoxic drugs in patients with malignant neoplasms. Figure 2 presents a decision tree developed by the authors to summarize the key considerations during the surgical implantation of implantable vascular access devices. The schematic outlines the clinical and technical factors influencing procedural planning and frames the central question addressed by this study.

Although no premature port removal occurred in this study. The overall implantation success rate defined as successful device placement with adequate catheter tip positioning exceeded 96.6% patients: 97.8% in the technology-assisted approach (TA) group and 98.8% in the anatomical landmark-based approach (ARP) group. This is considered a high success rate compared with similar studies, which reported success rates of 91.9% with an ARP approach and nearly 100% among patients who received ultrasound-guided implantation^9,10^.

Operative time was stratified using a 30-minute cutoff based on institutional standards commonly applied in Colombian surgical practice for TIVAD implantation. Procedures exceeding this duration may reflect increased technical complexity or intraoperative difficulty. This pragmatic threshold was used to explore the potential association between prolonged operative time and postoperative complications rather than to define procedural quality.

### Venous access selection

Certain conditions, such as burns or congenital and acquired morphological alterations, may impact the ability to identify anatomical landmarks^11^. Vascular access has been associated with complications, and these aspects have been evaluated in randomized controlled trials, in which the choice of vessel, laterality, as well as device caliber and length have been systematically analyzed^12^.

Meta-analyses have evaluated the risk of thrombosis associated with venous access, with no statistically significant differences found^13,14^. Other studies with lower statistical power have also failed to demonstrate the relevance of laterality in thrombosis risk^15^. The identification of a catheter associated with the innominate vein in the ARP group was an incidental finding during retrospective image review for catheter tip position assessment and does not reflect a routinely planned puncture technique for TIVAD implantation.

### Surgical approach

The use of anatomical landmarks has been reported to achieve a success rate of up to 87%. However, the use of technology-assisted devices appears to improve this success rate, approaching 100%^16^. Historically, surgeons relied on the subclavian access route, which is based on anatomical landmarks, for training purposes. Nevertheless, ultrasound-guided venipuncture has gained popularity and has demonstrated sufficient evidence to support its use. In contrast, venous dissection approaches are becoming increasingly unnecessary^17^.

Venous dissection was employed in a limited number of cases as a secondary strategy after unsuccessful initial percutaneous access attempts, rather than as a planned first-line approach for TIVAD implantation. Given the retrospective nature of the data collection, detailed intraoperative decision-making processes and the precise anatomical rationale for vessel selection could not be fully captured, which may introduce bias in the interpretation of these findings.

Previous failed insertion attempts, particularly in emergency settings, are among the most common predictors of insertion-related complications. It has been reported that with a single successful catheterization attempt, the complication rate is 4.3%, whereas this increases to 24% with two or more attempts^18,19^.

The identification of a catheter associated with the innominate vein in the ARP group was an incidental finding during the retrospective image review for catheter tip position assessment and does not reflect a routinely planned puncture technique for TIVAD implantation.

### Catheter tip positioning

Catheter tip location appears to be related to catheter dysfunction, manifesting as difficulty in aspiration, resistance, inflammation, and/or pain during infusion^20^. Additionally, catheter tip malposition is an independent risk factor for venous thrombosis in cancer patients, with a sevenfold increased risk when the catheter tip is located in the upper half of the superior vena cava^21^.

A noteworthy finding of this study was the heterogeneity observed in the interpretation of the final catheter tip location, as reported by both surgeons and radiologists. This finding suggests the need for additional studies to evaluate the degree of concordance in image interpretation.

Due to inconsistent documentation of the final catheter tip position across procedural reports, catheter tip location was retrospectively reassessed according to the Cadman classification by means of a systematic, patient-by-patient review of radiological studies. This assessment was performed by a radiologist to ensure data consistency and standardization.

### Use of prophylactic antibiotics

As intravascular devices, catheters may be associated with bacteremia-related complications. However, retrospective studies have reported a relative risk (RR) of 0.97, indicating that there is no association between infection and the use of prophylactic antibiotics. However, this recommendation lacks the statistical power to be considered a standard^22,23^.

Our findings indicate that the use of prophylactic antibiotics during catheter insertion did not confer a protective effect against infectious complications. During the 90-day follow-up period, antibiotic prophylaxis use was associated with a significantly higher risk of complications compared to no prophylaxis (RR 2.56; 95% CI 1.20–5.44). It cannot be determined whether the observed association between antibiotic use and complications reflects a true causal relationship or is attributable to confounding, particularly given that antibiotics may have been administered as prophylaxis for concomitant procedures that themselves could have increased the risk of complications.

Recently, the Society of Interventional Radiology classified TIVAD placement as a clean procedure according to the National Academy of Sciences/National Research Council recommendations, suggesting that prophylaxis should not be routinely recommended, regardless of the technique used^23,24^.

### Anesthesia management

The literature has not identified differences in terms of clinical outcomes, surgical time, blood loss, or radiation dose and duration. However, compared with local anesthesia, general anesthesia is associated with longer operating room times and increased costs^25^. In this cohort, the use of general anesthesia was not inherent to the TIVAD implantation technique but was instead dictated by the need to perform concomitant surgical procedures, including staging laparoscopies or oncologic resections. As such, anesthesia modality should be interpreted as a marker of overall surgical context rather than as a determinant of catheter implantation outcomes.

### Complications

Complications have been classified into those related to catheter insertion, those associated with port implantation, and those categorized on the basis of the time of occurrence^26^. Table 6 was describes a summary tool to provide an overview of the temporal occurrence of procedure-related complications, facilitating interpretation as a descriptive synthesis rather than an independent analytical component.

**Table 6:** Complications associated with implantable vascular access devices.

Early complications		Late complications	
Catheter insertion	REservoir implantation	Catheter insertion	Reservoir implantation
Pneumothorax	Dehiscence	Occlusion/Embolization	Rotation
Hematoma*	Hematoma*	Displacement	Extravasation
Arterial puncture	Seroma	Thrombosis	Membrane disruption
Hematoma	Surgical site infection	Catheter fracture	Pinch-off
–	–	Arrhythmia	–

* Hematoma is an early perioperative complication that may arise either during venous catheter insertion due to vascular injury or during the creation of the subcutaneous pocket for reservoir implantation.

Recent publications report overall complication rates ranging from 2% to 14.4%, which is consistent with the data presented in this study^10^. In our study, the absence of statistically significant differences in complication rates between the two implantation techniques is consistent with findings reported in the existing literature. No significant differences were observed in the incidence of either early or late complications between the groups, supporting comparable safety profiles for both approaches. It is important to note that all procedures were performed electively and predominantly by surgeons with extensive professional experience, factors that may have contributed to the overall low complication rates observed in our cohort^2^.

Venous thrombosis is a multifactorial condition and appears to be secondary to fibrin deposits due to endothelial injury, leading to a reduction in the vessel lumen^27^. Some studies have demonstrated a benefit of using the jugular vein to reduce risk, although other authors have reported no significant differences^28^. In our study, the single case of deep vein thrombosis occurred in a female patient with advanced gastric cancer, a condition associated with an increased thrombotic risk. The catheter was placed via a left internal jugular approach using anatomical landmarks, and retrospective imaging review demonstrated suboptimal tip positioning, suggesting a multifactorial etiology involving both patient-related and procedural factors.

Cardiac arrhythmias are among the potentially life-threatening complications associated with catheter tip migration, occurring in up to 9% of cases. However, evidence suggests no statistically significant differences in vascular access when subclavian vein access is compared with internal jugular vein access^29^.

Hemothorax is less common and has been more commonly associated with venous access in the right internal jugular and subclavian veins, particularly following arterial puncture or laceration of intrathoracic veins. Vascular injuries are uncommon. The incidence of internal carotid artery puncture is 3%, whereas subclavian artery puncture is reported even less frequently^26^.

Although it has a very low incidence rate, pneumothorax is the most common complication in patients with anatomical landmark-based placement [ARP]. Several studies, including expert consensus, have indicated that technology-assisted TIVAD insertion completely eliminates the risk of pneumothorax and hemothorax. However, pneumothorax is a significant complication following TIVAD insertion and has an immediate clinical impact. Its reported incidence varies between 0.5% and 6% and is highly associated with the ARP approach and multiple puncture attempts^30^. In our study, the incidence of this complication was lower in the technology-assisted group compared with the anatomical landmark-based group; however, this difference did not reach statistical significance (*P* = 0.17). Although this observation suggests a possible trend, the low event rate limits the statistical power of the analysis, and therefore no definitive conclusions can be drawn regarding a protective effect of the technology-assisted approach.

Among the complications associated with port implantation, wound dehiscence and leakage of contents may be related to technical errors in wound closure, improper positioning and fixation of the reservoir, and patient immunocompromised status and malnutrition. However, maintaining skin integrity and ensuring a tension-free incision reduce the incidence of these complications^26^.

Major complications (as classified by the Clavien–Dindo system) and rare events such as gas embolism, hemothorax, pericardial tamponade, and brachial plexus injury were not observed in our cohort. Currently, there are no established quality control standards for TIVAD implantation techniques or specific guidelines for managing complications. Although our study results indicate that the complication rate does not seem to correlate with operator experience, especially since the procedures were performed by both surgeons and interventional radiologists, several studies suggest that the level of experience of the physician inserting the TIVAD is crucial. The likelihood of complications is halved when the procedure is performed by a physician with experience in 50 or more insertions compared with a physician with fewer than 50 implantations^18,19^. No cases of catheter fracture or reservoir malposition were identified in our cohort.

The occurrence of hematoma was identified retrospectively from clinical records, without standardized documentation regarding size or severity, which may introduce reporting bias. Furthermore, technology-assisted techniques may entail increased tissue manipulation during image acquisition and needle adjustment, potentially contributing to the observed distribution of this complication.

### Medical specialty

The medical specialties currently involved in this study are limited to surgeons and interventional radiologists. However, the insertion procedure can also be performed by other specialties, including internal medicine, anesthesiology, and oncology. Nonetheless, the mastery of implantation techniques, complication management, and the appropriate use and maintenance of TIVADs remain inconsistent across different medical specialties. Furthermore, considering the low degree of participation of resident physicians reported in this study, there is a clear need to promote the development of these skills within specialized medical-surgical training programs.

The reduced involvement of resident physicians reflects the institutional characteristics of the participating centers, where formal training programs were not continuously available throughout the study period. Consequently, a proportion of procedures were performed exclusively by attending surgeons or radiologists, which should be considered when interpreting operator-related factors in this cohort.

### Limitations

Despite the multicenter design of the present study, the relatively low number of complications may have limited the statistical power to detect meaningful differences between implantation techniques. This observation may be partly attributable to selection bias, as most procedures were elective and performed by experienced operators in high-volume centers, potentially resulting in a lower overall complication rate. Although matched-pair or propensity-based analyses could further control for confounding, the retrospective nature of the study and the limited number of outcome events precluded the implementation of a robust matching strategy without substantially compromising sample size. These limitations underscore the need for adequately powered prospective controlled studies to validate the present findings.

Although current guidelines recommend positioning the catheter tip at the cavoatrial junction or within the superior vena cava, optimal positioning was not achieved in all patients in this cohort. This finding is likely multifactorial and may reflect variability in intraoperative verification methods, institutional practices, and historical changes in positioning criteria over the extended study period. In addition, final catheter tip location was retrospectively reassessed using standardized radiological criteria rather than real-time intraoperative confirmation, which may have contributed to discrepancies between guideline-defined optimal positioning and observed anatomical locations. Importantly, these findings should not be interpreted solely as a surrogate for technical proficiency, but rather as a reflection of real-world practice in a multicenter retrospective setting.

## Conclusions

The implantation of totally implantable venous access devices (TIVADs) in oncology patients with different primary tumor types has been demonstrated to be a safe and feasible procedure. Our study is the largest study conducted in Latin America and provides significant evidence for the optimization of insertion techniques and their ability to reduce complications.

The use of ultrasound-guided vascular access, combined with catheter tip position verification through electrocardiography (EKG), X-ray, or fluoroscopy, was associated with higher procedural success rates. Although a lower incidence of pneumothorax was observed in the technology-assisted group, this difference did not reach statistical significance, and no overall reduction in early complications was demonstrated.

Although this study provides a comprehensive assessment of the advantages and limitations of different TIVAD insertion techniques, reflecting their clinical applicability, further randomized controlled trials are recommended to validate these findings and definitively establish the optimal technique for TIVAD implantation.

## Data Availability

The datasets generated and analyzed during the present study are available for review upon reasonable request to the corresponding author. Due to the sensitive and confidential nature of the information, particularly as it involves personal health data, these datasets cannot be made publicly available in order to ensure compliance with ethical standards and data protection regulations.
